# Lactoferrin Attenuates Pro-Inflammatory Response and Promotes the Conversion into Neuronal Lineages in the Astrocytes

**DOI:** 10.3390/ijms26010405

**Published:** 2025-01-05

**Authors:** Melania Ruggiero, Antonia Cianciulli, Rosa Calvello, Dario Domenico Lofrumento, Concetta Saponaro, Francesca Martina Filannino, Chiara Porro, Maria Antonietta Panaro

**Affiliations:** 1 Department of Biosciences, Biotechnologies and Environment, University of Bari, 70125 Bari, Italy; melania.ruggiero@uniba.it (M.R.); antonia.cianciulli@uniba.it (A.C.); rosa.calvello@uniba.it (R.C.); 2 Department of Biological and Environmental Sciences and Technologies, Section of Human Anatomy, University of Salento, 73100 Lecce, Italy; dario.lofrumento@unisalento.it; 3 IRCCS Istituto Tumori Giovanni Paolo II, V.le O. Flacco, 65, 70124 Bari, Italy; c.saponaro@oncologico.bari.it; 4 Department of Clinical and Experimental Medicine, University of Foggia, 71121 Foggia, Italy; francesca.filannino@unifg.it (F.M.F.); chiara.porro@unifg.it (C.P.)

**Keywords:** astrocytes, lactoferrin, neuroinflammation, astrocytes reprogramming, astrocyte-to-neuron conversion, neurodegenerative diseases

## Abstract

Neurodegenerative diseases are characterized by progressive loss of neurons and persistent inflammation. Neurons are terminally differentiated cells, and lost neurons cannot be replaced since neurogenesis is restricted to only two neurogenic niches in the adult brain, whose neurogenic potential decreases with age. In this regard, the astrocytes reprogramming into neurons may represent a promising strategy for restoring the lost neurons and rebuilding neural circuits. To date, many anti-inflammatory agents have been shown to reduce neuroinflammation; however, their potential to restore neuronal loss was poorly investigated. This study investigates the anti-inflammatory effects of lactoferrin on DI-TNC1 astrocyte cell line and its ability to induce astrocyte reprogramming in a context of sustained inflammation. For this purpose, astrocytes were pre-treated with lactoferrin (4 μg/mL) for 24 h, then with lipopolysaccharide (LPS) (400 ng/mL), and examined 2, 9 and 16 days from treatment. The results demonstrate that lactoferrin attenuates astrocyte reactivity by reducing Toll-like receptor 4 (TLR4), Glial fibrillary acidic protein (GFAP) and IL-6 expression, as well as by upregulating Interleukin-10 (IL-10) cytokine and NRF2 expression. Moreover, lactoferrin promotes the reprogramming of reactive astrocytes into proliferative neuroblasts by inducing the overexpression of the Sex determining region Y/SRY-box 2 (SOX2) reprogramming transcription factor. Overall, this study highlights the potential effects of lactoferrin to attenuate neuroinflammation and improve neurogenesis, suggesting a future strategy for the treatment of neurodegenerative disorders.

## 1. Introduction

Neuroinflammation is a defence mechanism aiming to protect the brain from harmful stimuli generated by injuries or infections. Neuroinflammation spontaneously resolves with the elimination of pathogens, the clearance of cellular debris and tissue repair [1]. However, inflammatory responses can persist due to endogenous or environmental factors, ending up being detrimental [2]. Sustained inflammation fuels pro-inflammatory responses through a positive feedback mechanism potentially leading to neuronal damage and cell death.

Neurodegenerative diseases are characterized by progressive loss of neurons in the central or peripheral nervous system. The loss of neurons affects the functionality of neural circuits culminating in impaired memory, cognition, behaviour, sensory and/or motor functions [3]. Neurons are terminally differentiated cells and neurogenesis is restricted to only two neurogenic niches in the adult brain, the subventricular zone and the dentate gyrus [3,4]. In aged individuals, the most affected by neurodegenerative disorders, the neurogenic potential is compromised; thus, the age-related neuron loss cannot be properly solved.

Neuroinflammation is involved in the pathogenesis of neurodegenerative diseases by contributing to neuron loss. In this light, anti-inflammatory agents were utilized in the therapies for slowing down the progression of neurodegenerative diseases [5]. Although many anti-inflammatory agents have been shown to exert neuroprotective effects showing promising results [6,7,8,9,10,11], only curcumin was able to resolve the neuron loss by improving neurogenesis in an Alzheimer’s disease (AD) mouse model [12].

Cell reprogramming is a strategy based on the conversion of somatic cells into cell type of another specific lineage [13]. Due to the limited regeneration abilities of the central nervous system (CNS), cell reprogramming could represent a promising strategy for compensating the loss of neurons that occurs in neurodegenerative diseases. Astrocytes are an optimal source of potentially reprogrammable cells, since they are widely distributed into the CNS, share common progenitors with neurons and have an inherent proliferative capacity [14]. In addition, astrocytes possess an intrinsic and latent neurogenic program that can be activated in a pro-inflammatory environment [15]. Thus, in response to immunologic challenges or brain injuries, reactive astrocytes are activated [16] and acquire neurogenic potential, besides participating in the inflammatory responses by releasing inflammatory mediators and cytokines [17]. On the other hand, reactive astrocytes can also inhibit neural regeneration through the glial scar formation [18]. Therefore, controlling reactive astrogliosis is essential for preventing brain damage and promoting brain repair [14].

Astrocyte reprogramming into neurons can be achieved by modulating the activity of several transcription factors and miRNAs [19]. Specifically, the overexpression of a single transcription factor, Sex determining region Y/SRY-box 2 (SOX2), has been shown to reprogram the astrocytes into proliferating neuroblasts [20] that can differentiate into mature neurons when supplied with neurotrophic factors [21].

Lactoferrin is a glycoprotein commonly found in the exocrine fluids, such as saliva, tears, nasal and bronchial secretions, vaginal and seminal fluids and gastrointestinal fluids, and particularly in milk and colostrum [22,23,24]. Lactoferrin exists also in secondary granules of neutrophils and is released in response to inflammatory stimuli [22,23,24]. Many functions have been attributed to lactoferrin, such as anti-inflammatory, antibacterial, antiviral, antioxidant and immunomodulatory ones [25]. Lactoferrin was found to be abnormally expressed in the lesion areas of different neurodegenerative diseases, suggesting its potential activity in promoting neuroprotection and neurogenesis [26,27].

The present study aimed to investigate the ability of human lactoferrin in modulating astrocyte reactivity by using the Lipopolysaccharide (LPS)-induced DI-TNC1 cell line as a model of neuroinflammation [28]. In addition, the lactoferrin effects on the astrocytes reprogramming into neurons under permissive conditions of prolonged inflammation were investigated.

## 2. Results

### 2.1. Effects of Lactoferrin on Viability of DI-TNC1 Cells

**Figure 1:**
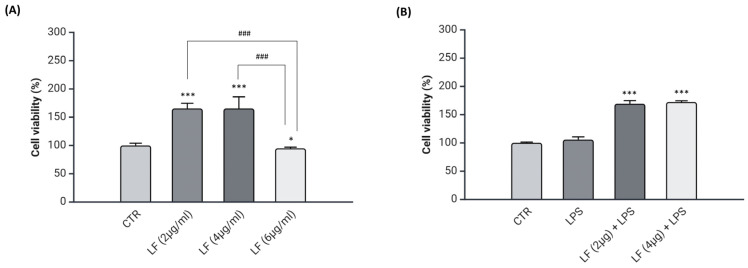
Viability analysis of DI-TNC1 cells treated with lactoferrin. MTT assay was performed after treating the cells with lactoferrin (2–6 μg/mL) for 72 h (**A**). In (**B**), cells were pre-treated with lactoferrin (2 μg/mL or 4 µg/mL) for 24 h followed by LPS (400 ng/mL) for 48 h, or treated with LPS alone for 48 h. Data are presented as means ± SD of triplicate measurements from three independent experiments. * *p* < 0.05 vs. CTR; *** *p* < 0.001 vs. CTR; ### *p* < 0.001 vs. LF (2 μg/mL) or LF (4 μg/mL).

Preliminary studies on lactoferrin cytotoxicity against DI-TNC1 cells were performed by MTT assay. Cells were treated with lactoferrin concentrations ranging from 2 µg/mL to 6 µg/mL for 72 h. Figure 1A shows that the treatment with lactoferrin 2 μg/mL or 4 μg/mL increased significantly the number of viable cells compared to the control. Due to the significant reduction of cell viability following the treatment with lactoferrin 6 μg/mL compared to the control (CTR), this concentration was excluded. Next, to evaluate the combined effects of lactoferrin and LPS on DI-TNC1 cell viability, lactoferrin (2 μg/mL or 4 μg/mL) was tested in association with LPS (Figure 1B). For this purpose, DI-TNC1 cells were pre-treated with lactoferrin for 24 h and stimulated with LPS 400 ng/mL for 48 h or treated with LPS alone for 48 h. Previous works showed that the concentration of LPS 400 ng/mL is enough to induce the astrocyte activation [29,30]. Figure 1B shows that cell viability of pre-treated cells increased significantly compared to the control, suggesting that tested concentrations do not have cytotoxic effects when used either alone or in combination with LPS.

### 2.2. Lactoferrin Exerts Anti-Inflammatory Functions in the Astrocytes

**Figure 2:**
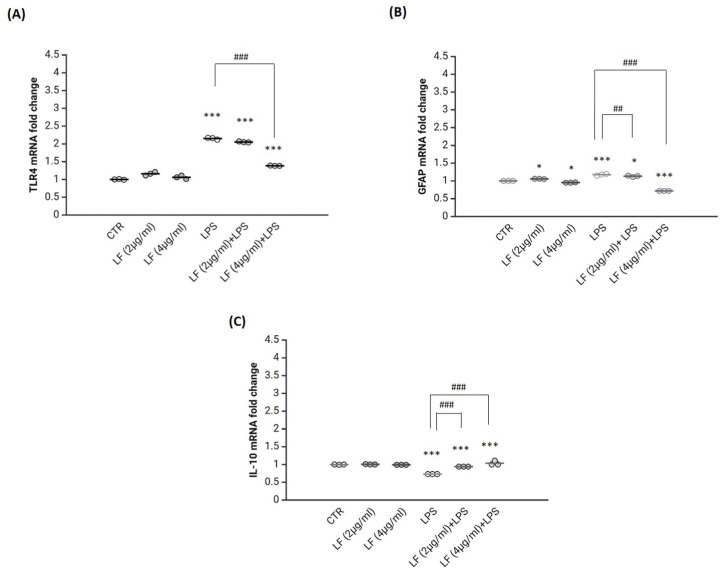
Anti-inflammatory effects of lactoferrin on DI-TNC1 cells. Real-time PCR analysis of TLR4 (**A**), GFAP (**B**) and IL-10 (**C**) was performed after the treatments with lactoferrin, LPS alone (400 ng/mL) and with lactoferrin plus LPS for 48 h. Untreated cells were used as control. GAPDH was used as a housekeeping gene for normalization. Results are presented as means ± SD of triplicate measurements from three independent experiments. * *p* < 0.05 vs. CTR; *** *p* < 0.001 vs. CTR; ## *p* < 0.01 vs. LPS; ### *p* < 0.001 vs. LPS.

To test the possible anti-inflammatory effects of lactoferrin (2 or 4 µg/mL) on DI-TNC1 cells, the expression levels of Toll-like receptor 4 (TLR4) (Figure 2A) as pro-inflammatory marker, Glial fibrillary acidic protein (GFAP) (Figure 2B) as astroglial reactivity marker and Interleukin-10 (IL-10) as anti-inflammatory marker (Figure 2C) were examined after 2 days post-treatment (dpt) according to preliminary experiments and as previously reported [31]. As expected, the astrocytes were activated by the stimulation with LPS showing a pro-inflammatory phenotype, characterized by significantly higher levels of TLR4 and GFAP and lower levels of IL-10 in comparison to the untreated cells. However, as shown in Figure 2A, when activated cells were pre-treated with lactoferrin 4 µg/mL, the TLR4 expression was strongly reduced. In contrast, 2 µg/mL of lactoferrin were unable to overcome the LPS-induced effects on TLR4 expression. Lactoferrin was also effective in modulating the GFAP expression (Figure 2B). Indeed, both concentrations of lactoferrin were able to reduce the expression of GFAP in the presence of LPS compared to the LPS group, although the concentration of 4 µg/mL was found to be more effective in counteracting the LPS-induced astrocyte reactivity. Apart from reducing the expression of pro-inflammatory markers, the pre-treatment with lactoferrin (2 or 4 µg/mL) significantly increased the expression of the IL-10 anti-inflammatory cytokine in comparison to the LPS treatment alone, with the higher concentration being more effective.

**Figure 3:**
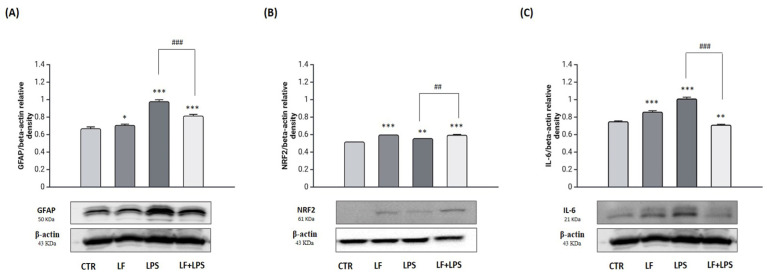
Anti-inflammatory effects of lactoferrin on the protein levels in DI-TNC1 cells. Western blot analysis of GFAP (**A**), NRF2 (**B**) and IL-6 (**C**) was performed after the treatments with lactoferrin, LPS alone (400 ng/mL) and with lactoferrin plus LPS for 48 h. Untreated cells were used as control (CTR). β-actin was used as a housekeeping gene for normalization. Results are presented as means ± SD of triplicate measurements from three independent experiments. * *p* < 0.05 vs. CTR; ** *p* < 0.01 vs. CTR; *** *p* < 0.001 vs. CTR; ## *p* < 0.01 vs. LPS; ### *p* < 0.001 vs. LPS.

Overall, the concentration of 4 µg/mL showed better outcomes in reducing astrocytes’ reactivity. To confirm the results obtained with real-time polymerase chain reaction (RT-PCR), the anti-inflammatory effects of lactoferrin 4 µg/mL were tested on the protein level (Figure 3).

Western blotting analysis proved that lactoferrin 4 µg/mL downregulates the expression of GFAP protein in reactive astrocytes compared to the only LPS-treatment, confirming the regulation of GFAP expression both at the transcriptional and translational level (Figure 3A). Reactive astrocytes exhibited lower levels of the NRF2 protein, a transcription factor involved in the regulation of antioxidant and anti-inflammatory responses [32]. Figure 3B shows that NRF2 was significantly upregulated by lactoferrin pre-treatment compared to the LPS-treatment. The expression levels of the IL-6 pro-inflammatory cytokine were also tested (Figure 3C). LPS-treated cells significantly upregulate the expression of the IL-6 cytokine compared to the control. In contrast, when reactive astrocytes were pre-treated with lactoferrin, they strongly downregulate the expression of the IL-6 pro-inflammatory cytokine.

### 2.3. Lactoferrin Triggers the SOX2 Reprogramming Transcription Factor by Inducing a Proliferative State

**Figure 4:**
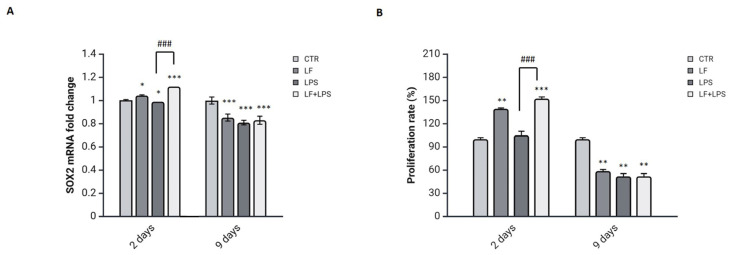
Analysis of SOX2 expression and proliferation rate. Cells were pre-treated with lactoferrin for 24 h followed by LPS for 48 h or, alternatively, with LPS alone for 48 h or lactoferrin alone for 72 h. The SOX2 mRNA expression levels were examined from 2 to 9 dpt (**A**). Cell proliferation rate of DI-TNC1 cells was assessed from 2 to 9 dpt (**B**). The percentages of viable cells were calculated by comparison to untreated viable cells taken as control. Results of Real-time PCR and MTT assay were expressed as mean ± SD of 3 measurements (n = 5 experiments). * *p* < 0.05 vs. CTR; ** *p* < 0.01 vs. CTR; *** *p* < 0.001 vs. CTR; ### *p* < 0.001 vs. LPS.

To analyze the effects of lactoferrin on the astrocytes reprogramming, we examined the expression of SOX2, a transcription factor involved in the reprogramming process [20]. The expression levels of SOX2 were evaluated at the transcriptional level at 2 and 9 dpt (Figure 4A).

As shown in Figure 4A, while the LPS induction decreased the SOX2 endogenous levels compared to the control, the pre-treatment with lactoferrin strongly elicits the SOX2 expression at 2 dpt in comparison to untreated and LPS-treated cells. However, the SOX2 expression levels were significantly reduced after 9 days in response to the LF+LPS treatment compared to the untreated cells.

SOX2 can reprogram astrocytes into neuroblasts with high proliferative activity [33]. Thus, we assessed cell proliferative activity of DI-TNC1 cells from 2 to 9 dpt by performing the MTT assay (Figure 4B). Remarkably, the proliferation rate was greatly improved by the LF+LPS treatment at 2 dpt and then decreased after 9 days from treatment compared to the untreated cells, following the same trend of the SOX2 expression.

### 2.4. Lactoferrin Stimulates the Reprogramming of Reactive Astrocytes into Neuroblasts

**Figure 5:**
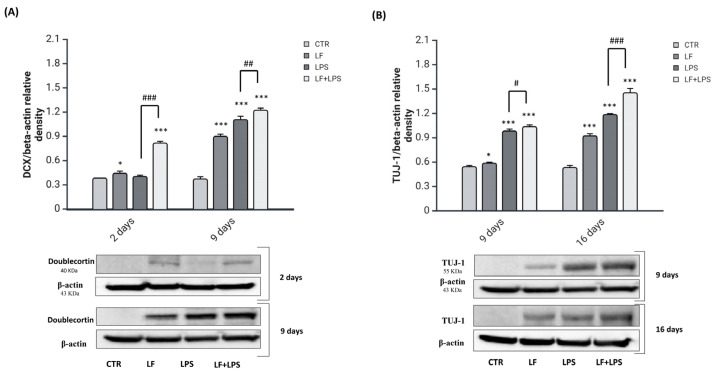
Analysis of neuronal reprogramming markers. Immunodetection of DCX (**A**) and TUJ-1 (**B**) in untreated (CTR), lactoferrin-treated (LF), LPS-treated (LPS) and lactoferrin plus LPS-treated (LF+LPS) cells from 2 to 16 dpt. Results of densitometric analysis were normalized to β-actin as housekeeper gene and expressed as mean ± SD of 3 measurements (n = 5 experiments). * *p* < 0.05 vs. CTR; *** *p* < 0.001 vs. CTR; # *p* < 0.05 vs. LPS, ## *p* < 0.01 vs. LPS; ### *p* < 0.001 vs. LPS.

To test if lactoferrin has effects on the conversion of reactive astrocytes into neuroblasts, we assessed the expression of doublecortin (DCX) as a specific marker of neuroblasts [34] at 2 and 9 dpt (Figure 5A). Then to follow the differentiation of neuroblasts in post-mitotic neurons, the expression of Neuron-specific class III beta-tubulin (TUJ-1), a marker of early post-mitotic neurons [35], was investigated from 9 to 16 days post-treatment (Figure 5B).

In response to the LPS alone, reactive astrocytes did not exhibit changes in doublecortin expression at 2 dpt in comparison to the control. The scenario changed after 9 days, when LPS-treated cells strongly increased the expression of DCX compared to the untreated cells. Surprisingly, when reactive astrocytes were pre-treated with lactoferrin, doublecortin expression was highly increased as early as 2 dpt. After 9 days, the expression of DCX was strongly enhanced by the LF pre-treatment compared to the LPS alone treatment. As shown in Figure 5B, LPS-induced cells activated the expression of TUJ-1 at 9 and 16 days post-treatment. However, cells subjected to the combined treatment increased the expression of the early post-mitotic neuron marker in comparison to the LPS treatment both at 9 and 16 dpt.

**Figure 6:**
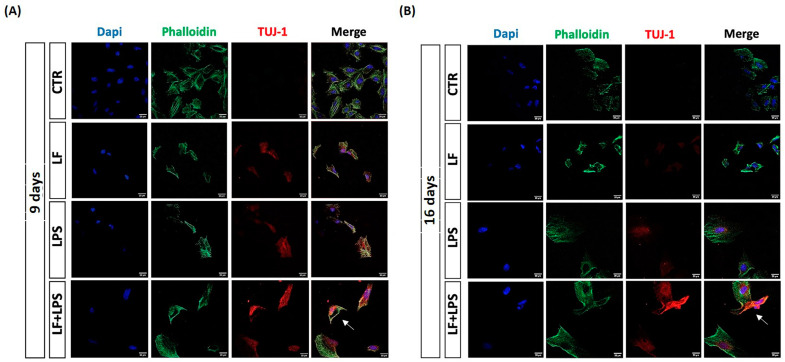
Double immunostaining for TUJ-1 and phalloidin at 9 dpt (**A**) and 16 dpt (**B**). Green (AlexaFluor488) = phalloidin, red (TRITC) = TUJ-1, blue (DAPI) = nuclei. Scale bar = 20 µm. Cells with a neural precursor-like morphology are indicated by the with arrows.

TUJ-1 expression was also evaluated by immunofluorescence analysis from 9 dpt (Figure 6).

Figure 6 shows that the immunostaining for TUJ-1 was more intense in cells pre-treated with lactoferrin in comparison to the LPS-induced cells at 9 dpt (Figure 6A) and 16 dpt (Figure 6B), in accordance with the immunoblotting analysis. Despite the TUJ-1 expression, cells treated with LPS alone did not acquire a neuron-like morphology at 9 and 16 dpt. However, in response to the pre-treatment with lactoferrin, some TUJ-1 positive cells displayed a neuroblast-like morphology (as indicated by the white arrow in Figure 6A) at 9 dpt and acquire an elongated morphology (as indicated by the white arrow in Figure 6B) after 16 days.

### 2.5. Lactoferrin Induces the Acquisition of Neuronal Features

**Figure 7:**
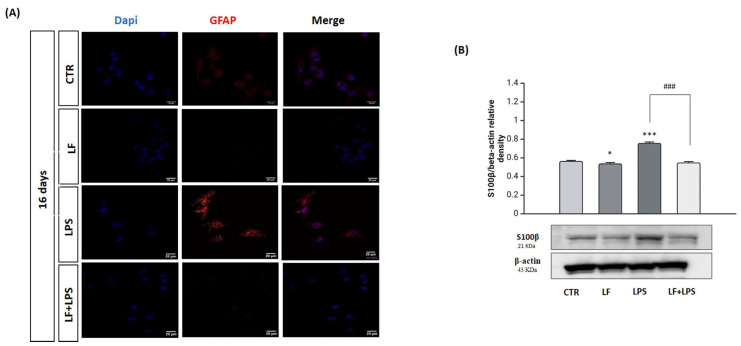
Analysis of astrocyte specific markers. Immunostaining for GFAP (**A**) at 16 dpt. Red (TRITC) = GFAP, blue (DAPI) = nuclei. Scale bar = 20 µm. Immunodetection of S100β (**B**) in untreated (CTR), lactoferrin-treated (LF), LPS-treated (LPS) and lactoferrin plus LPS-treated (LF+LPS) at 16 dpt. Results of densitometric analysis were normalized to β-actin as housekeeper gene and expressed as mean ± SD of 3 measurements (n = 5 experiments). * *p* < 0.05 vs. CTR; *** *p* < 0.001 vs. CTR; ### *p* < 0.001 vs. LPS.

The conversion of reactive astrocytes into neurons involves the loss of the astrocyte-specific phenotype and the gaining of neuronal properties [36]. To assess the loss of astrocytes features, we performed immunofluorescence analysis for detecting GFAP and Western blotting to detect the S100β protein as astrocyte-specific markers at 16 dpt (Figure 7).

Figure 7A shows that lactoferrin pre-treatment strongly reduced the number of GFAP positive cells in comparison to the LPS treatment alone and controls. In addition, lactoferrin pre-treated cells significantly reduced the expression of S100β protein compared to the LPS-treated cells (Figure 7B).

**Figure 8:**
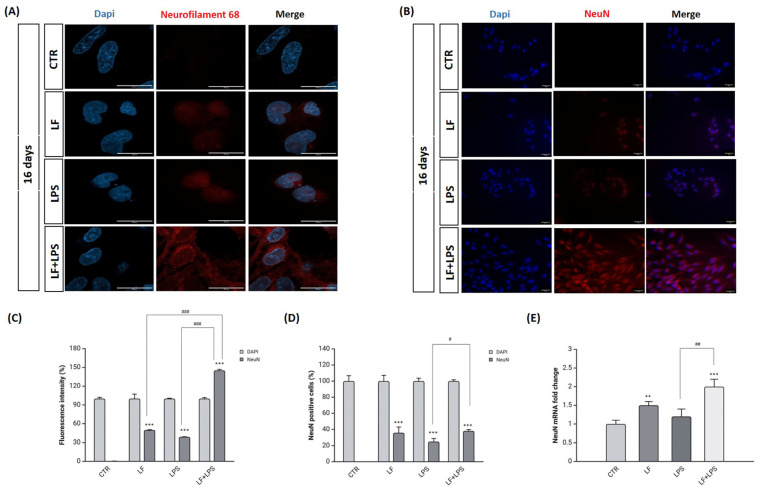
Analysis of neuronal markers. Immunostaining for Neurofilament 68 (**A**) at 16 dpt. Red (TRITC) = Neurofilament 68, blue (DAPI) = nuclei. Scale bar = 20 µm. Immunostaining for NeuN (**B**) at 16 dpt. Red (TRITC) = NeuN, blue (DAPI) = nuclei. Scale bar = 200 µm. Analysis of fold change in fluorescence intensity (**C**). The area of interest was selected to measure the relative fluorescence intensity. Data are expressed as percentage of respective controls. Results are expressed as mean ± SD of 3 measurements (n = 5 experiments). *** *p* < 0.001 vs. CTR; ### *p* < 0.001 vs. LF; ### *p* < 0.001 vs. LPS. Number of NeuN positive cells per field of view (**D**). Population of NeuN^+^ cells versus DAPI^+^ cells expressed as percentage. Only cells stained exclusively within nuclei for NeuN were counted. Results are expressed as mean ± SD of 3 measurements (n = 5 experiments). *** *p* < 0.001 vs. CTR; # *p* < 0.01 vs. LPS. Real-time PCR analysis of NeuN (**E**) was performed after the treatments with lactoferrin, LPS alone and with lactoferrin plus LPS for 16 days. Untreated cells were used as control. GAPDH was used as a housekeeping gene for normalization. Results are presented as means ± SD of triplicate measurements from three independent experiments. ** *p* < 0.01 vs. CTR; *** *p* < 0.001 vs. CTR; ## *p* < 0.001 vs. LPS.

To assess the gaining of neuronal features, moreover, we performed immunofluorescence analysis for detecting Neurofilament 68 (NF) and Neuronal nuclei (NeuN) as neuronal markers (Figure 8).

The immunostaining for NF68 reveals that, following LPS treatment, the neuronal protein is mainly localized in the nuclear or perinuclear space (Figure 8A). However, following combined treatment, NF68 relative red staining spreads to form processes extending from the cell body (Figure 8A). Moreover, the immunostaining for NeuN reveals that NeuN was expressed by lactoferrin pre-treated cells to a greater extent compared to LPS-treated cells, as also showed by fluorescence intensity analysis (Figure 8B,C). These results were also confirmed by the number of NeuN positive cells as reported in Figure 8D that reports the percentage of cells stained exclusively within nuclei for NeuN. Further confirmation was obtained by quantitatively analyzing NeuN expression at the transcriptional level (Figure 8E).

### 2.6. Tumorigenicity Assessment for Reprogrammed Cells

**Figure 9:**
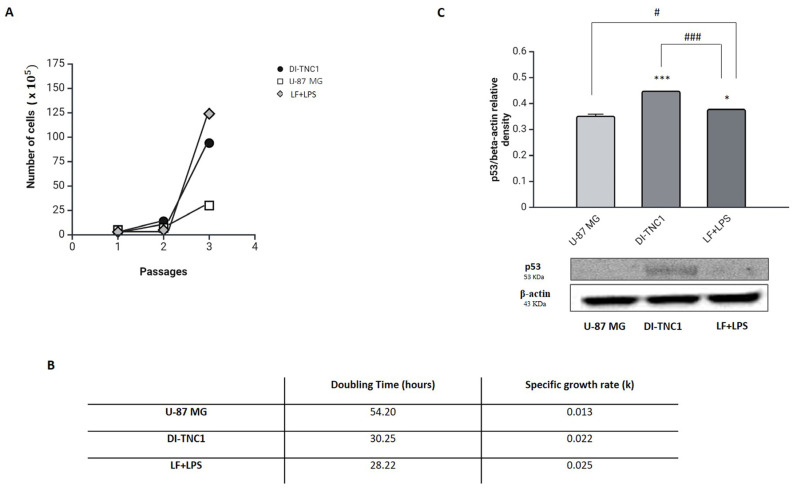
Cell growth curve (**A**), doubling time and specific growth rate (**B**) of U-87 MG, DI-TNC1 and DI-TNC1 LF+LPS-treated cells. Immunodetection of p53 (**C**) in U-87 MG, DI-TNC1 and DI-TNC1 LF+LPS-treated cells. Results of densitometric analysis were normalized to β-actin as housekeeper gene and expressed as mean ± SD of 3 measurements (n = 5 experiments). * *p* < 0.05 vs. unstimulated cells; *** *p* < 0.001 vs. unstimulated cells; # *p* < 0.01 vs. LF+LPS; ### *p* < 0.001 vs. LF+LPS.

The overexpression of SOX2 has been reported in several tumors, including glioblastoma [37]. To verify the tumorigenic potential of reprogrammed cells, we performed a proliferation rate assay to compare the cell growth properties of lactoferrin pre-treated cells with those of U-87 MG glioblastoma cell line (Figure 9A,B).

From proliferation rate assay emerged that the growth rate of DI-TNC1 cells treated with LF+LPS greatly differ from the U-87 MG one, as shown in Figure 9A. This observation was strongly supported by the values of doubling time, expressed in hours, and specific growth rate (Figure 9B), calculated as described in the Material and Methods section.

To further analyze the tumorigenic potential of double-treated cells, we assessed the expression levels of the tumor suppressor protein p53 (Figure 9C). Surprisingly, Western blot analysis revealed that DI-TNC1 pre-treated with lactoferrin significantly decreased the expression of p53 compared to controls.

## 3. Discussion

The iron-binding protein lactoferrin enters the brain since it can easily cross the blood–brain barrier via receptor-mediated transcytosis [38,39]. In addition, lactoferrin is locally released by macrophages and astrocytes in response to inflammatory triggers [40,41]. The release of lactoferrin by reactive microglia and astrocytes, besides the increased levels of lactoferrin found in rat brains of Parkinson’s disease (PD) models, suggests a role in neuroprotection [42]. In support of this, lactoferrin is found to protect the brain from iron dysregulation and oxidative stress [43], beyond slowing the progression of the disease by protecting dopaminergic neurons from neurodegeneration [44] and relieving motor deficits in PD mouse model [45]. Importantly, lactoferrin has well-known anti-inflammatory properties [46], and it is also positively associated to neurogenesis [47]. Indeed, lactoferrin is found to stimulate the development of immature neurons in a mouse model after acute gamma irradiation [48] and the growth of new neurons in a rat model of hepatic encephalopathy [27].

Neurodegenerative disorders are characterized by chronic inflammation and progressive loss of neurons. Therefore, anti-inflammatory agents, together with somatic cell reprogramming into neurons, have been proposed as therapeutic approaches [14,49]. Notably, astrocytes are the ideal candidates for conversion into neurons, due to their intrinsic neurogenic potential that can be unlocked in response to inflammatory stimuli [14,50,51].

In this work, we put together for the first time the neurogenic properties of lactoferrin with the reprogramming ability of reactive astrocytes. In addition, the anti-inflammatory properties of lactoferrin on the astrocytes were also tested. For this purpose, we used the DI-TNC1 cell line stimulated with LPS for 16 days as in vitro model of chronic neuroinflammation.

In response to inflammatory triggers, such as LPS, astrocytes are activated via the TLR4 signaling leading to the expression of pro-inflammatory genes and the upregulation of GFAP, a well-known reactive astrocyte marker [52,53]. As expected, we found that the LPS-treatment increased the expression of TLR4 and GFAP and decreased the expression of the NRF2 transcription factor, leading to the upregulation of the pro-inflammatory IL-6 cytokine and the downregulation of the anti-inflammatory IL-10 cytokine. Intriguingly, the pre-treatment with lactoferrin downregulated pro-inflammatory markers and upregulated the anti-inflammatory ones, showing that lactoferrin has the ability to reverse the LPS effects exerting anti-inflammatory effects.

The forced expression of SOX2 neural progenitor marker [33] in mature astrocytes has been found to drive the reprogramming into neural progenitors and functional neurons [20,21,54,55]. Interestingly, in this work we demonstrated that the pre-treatment with lactoferrin in reactive astrocytes boosts the SOX2 expression after 2 days post-treatment. Supporting the results that SOX2-induced reprogramming of mature astrocytes passes through proliferative neuroblasts [21,55], we found, in astrocyte cell cultures, that lactoferrin is able to increase the cell proliferation rate at 2 days post-treatment, besides enabling the expression of doublecortin, a known marker for neuroblasts [34,56]. These findings suggest that reactive DI-TNC1 cells pre-treated with lactoferrin are converted into proliferative neuroblasts as early as 2 days post-treatment via the forced expression of SOX2.

During the conversion of somatic cells into neurons, only some cells are successfully reprogrammed into neurons while others undergo cell death, due to a metabolic checkpoint [57]. Consistently, after 9 days we found a transient reduction of proliferation rate, besides the reduction of SOX2 expression. However, after 9 days, doublecortin is still expressed and it is also activated the expression of TUJ-1 protein, a marker of differentiating immature neurons [26] in lactoferrin pre-treated cells.

On the other hand, we found that the stimulation with LPS alone activates the latent neurogenic program of the astrocytes in a SOX2-independent manner, as demonstrated by the expression of doublecortin and TUJ-1 from 9 days post-treatment. However, despite the expression of neural markers, cells treated with LPS do not display a neural precursor-like morphology, as confirmed by the absence of neural processes extending from the cell body. In addition, LPS-treated cells maintained the expression of the astrocyte marker GFAP and upregulate the expression of the S100β protein at 16 days post-treatment. Collectively, these data suggest that the latent neurogenic program in the astrocytes is activated by LPS induction, but the conversion into neuron precursors is unsuccessful or, probably, requires more time. These limitations can be overcome by the pre-treatment with lactoferrin, which strongly reduces GFAP and S100β expression and activates the latent neurogenic program via SOX2 overexpression, leading reprogrammed cells to acquire a neural precursor-like morphology.

In this respect, astrocytes are primarily known for their roles in supporting neuronal function, maintaining the blood–brain barrier and modulating synaptic activity. However, recent studies have suggested that astrocytes may possess a latent plasticity, potentially transitioning into neurons under certain conditions. The mechanisms that drive astrocyte reprogramming remain a subject of intense investigation. One area of interest is whether astrocytes directly differentiate into neurons or whether they first shift through an intermediate neural stem/progenitor cell (NSC/NPC) state [58,59].

It is well known that lactoferrin has neuroprotective properties, particularly in response to oxidative stress and inflammation [26,60]. Given these effects, we may hypothesize that lactoferrin could facilitate the conversion of astrocytes into neurons, but it remains unclear whether this process occurs through a direct reprogramming mechanism or requires an intermediate NSC/NPC.

The findings presented in this study provide novel insights into the potential of astrocytes to transdifferentiate into neuronal cells following lactoferrin treatment.

The precise mechanisms underlying lactoferrin-induced neuronal differentiation remain to be fully elucidated. However, several potential pathways may be involved. Lactoferrin has been shown to possess immunomodulatory properties, and it is possible that it exerts its effects on astrocytes through the activation of specific signaling pathways. In this context, it was reported that lactoferrin is able to protect neurons from oxidative-stress-induced damage by decreasing apoptosis and by reducing astrocyte reactivity and so reducing brain inflammation response [61]. In addition, it was also reported that lactoferrin is able promote the survival of neurons in culture via activation of p38 MAPK [62].

Our data suggests that lactoferrin treatment may induce astrocytes to enter a more progenitor-like state. This is, in part, supported by the observed upregulation of stem cell markers such as SOX2 in astrocyte cultures following lactoferrin treatment, and by an increase in NeuN positive cells through a fine balancing of the astrocyte response to lactoferrin treatment. These findings are consistent with previous studies demonstrating that astrocytes can retain a degree of plasticity and reacquire stem cell properties under specific conditions [63]. However, our results undoubtedly need to be further explored in the future to clarify whether astrocytes convert into neurons/neuronal lineages directly or via the state of neural stem/progenitor cells after lactoferrin treatment.

The overexpression of SOX2, moreover, is also associated to several kinds of tumors, including glioblastoma [64]. Although lactoferrin pre-treated cells overexpress SOX2, we found that the reprogrammed cells differ in proliferation rate from the U-87 glioblastoma cell line, having a different doubling time as well as a specific growth rate. Surprisingly, we found that lactoferrin pre-treated cells almost silence the p53 transcription factor, a known tumor suppressor protein. However, the p53 signaling is also reported to be a critical checkpoint for the SOX2-mediated astrocytes reprogramming, and the silencing of p53 greatly boosts the production of induced adult neuroblasts [65]. In the light of these findings, the p53 silencing of lactoferrin pre-treated cells may be considered a strategy for improving the overall production of neuroblasts.

Furthermore, in addition to these observations, it emerges from recent studies the lactoferrin involvement in regulating cholesterol and lipid metabolism [66]. Dysregulated lipid metabolism can trigger inflammation, which is, as said, a key factor in neurodegenerative diseases, including AD [67,68].

Lactoferrin, as reported, seems to be implicated in peripheral cholesterol metabolism disorders. It has emerged, in fact, that the distribution of lactoferrin appears to change in astrocytes of aging brains and those exhibiting neurodegeneration [69].

As we report in the manuscript, lactoferrin has strong anti-inflammatory properties that, together with the evidence of its ability to reprogram astrocytes into neuronal precursors, could potentially mitigate damage related to neuroinflammation and neurodegeneration including those related to age [70]. In this respect, exploring the relationship between lactoferrin and lipid metabolism could be useful to identify new therapeutic targets for neurodegenerative diseases.

Overall, this study, although limited to its initial observation, demonstrates for the first time that lactoferrin reduces astroglial reactivity and leads to a modulation of markers of neuronal precursor differentiation (such as SOX2 and NeuN) in lactoferrin-treated astrocytes. Future studies are certainly needed to examine whether lactoferrin-induced neuroblasts can mature into functional neurons to resolve neuronal loss due to neurodegenerative diseases. Moreover, the present study was conducted using in vitro cell culture models, which may not fully reproduce the complex in vivo environment. Further studies are necessary in order to investigate the effects of lactoferrin on astrocyte-to-neuron conversion in animal models of neurodegeneration.

## 4. Materials and Methods

### 4.1. Cell Culture and Treatments

Rat type I astrocytes DI-TNC1 cell line was cultured in Dulbecco’s Modified Eagle Medium (DMEM) high-glucose supplemented with 10% Fetal Bovine Serum (FBS), 1% Penicillin–Streptomycin (Pen-Strep) Solution (Gibco-Thermo Fisher Scientific, Waltham, MA, USA) and 1% Glutammine (Sigma-Aldrich, St. Louis, MO, USA) and kept growing until 80% confluence at 37 °C in a humidified incubator with 5% ${CO}_{2}$. For cell treatments, lactoferrin from human milk (Sigma Aldrich, St. Louis, MO, USA) (4 µg/mL) and LPS from *E. coli* (400 ng/mL) (Sigma Aldrich, St. Louis, MO, USA) were used. DI-TNC1 cells were pre-treated with lactoferrin for 24 h and stimulated with LPS for 48 h or were treated with lactoferrin alone for 72 h or LPS alone for 48 h. Cells were continuously stimulated by refreshing the treatment medium every 3 days and were analyzed after 2, 9 and 16 days post-treatment.

For the MTT assay, the astrocytes were treated with lactoferrin alone at a concentration of 2 µg/mL, 4 µg/mL or 6 µg/mL for 72 h. In addition, cells were subjected to a combined treatment by pre-treating with lactoferrin (2 µg/mL or 4 µg/mL) for 24 h and with LPS for 48 h or were subjected to a single treatment with LPS alone for 48 h.

### 4.2. MTT Assay

Cytotoxic effects of lactoferrin on DI-TNCI cells were examined by the MTT (3-(4,5-Dimethylthiazol-2-yl)-2,5-Diphenyltetrazolium Bromide) assay. A total of 2.0 × $10^{4}$ cells were plated in 96-well plate and subjected to treatments. After 48 h, the MTT was added at a concentration of 1 mg/mL and cells were kept in a humidified ${CO}_{2}$ incubator at 37 °C for 4 h. The formazan crystals were solubilized in ethanol. Cell viability was quantified by reading the optical density at 540 nm on Cytation 3 Cell Imaging Multi-Mode Reader (Biotek, Winooski, VT, USA). Values were subtracted to the background measured at 690 nm and expressed as the average percentage ± SD.

### 4.3. Cell Lysates and Western Blotting

Cells were harvested and lysed with RIPA buffer containing 50 mM Tris pH 8, 1% Triton-X, 0.1% sodium dodecyl sulphate (SDS), 1.5 M NaCl plus protease inhibitors Aprotinin (4 U/mL), Leupeptin (1 µM), and PMSF (100 µM). After multiple freezing and thawing cycles, lysates were centrifuged at 12,000× *g* 30 min at 4 °C. The concentration of proteins was determined by the Bradford protein assay, measuring the absorbance at 595 nm and interpolating the optical density with a standard curve. For each treatment were uploaded 20 µg of proteins on 4–12% precast polyacrylamide gels, and the electrophoretic run was performed by applying a voltage of 200 V. The protein bands were transferred on nitrocellulose membranes and the target proteins were recognized by anti-doublecortin mouse polyclonal antibody (PoAb), anti-β3 tubulin (Tuj1) mouse monoclonal antibody (MoAb), anti-β actin mouse MoAb (1:500), anti-GFAP mouse MoAb, anti-NRF2 mouse MoAb, anti-IL6 goat PoAb (all from Santa Cruz Biotechnology, Inc., Milan, Italy) and anti-p53 mouse MoAb, anti-S100β mouse (1:500) (Sigma-Aldrich, St. Louis, MO, USA). For immunodetection, were used HRP-conjugated secondary antibodies (1:10,000) (Santa Cruz Biotechnology, Inc., Milan, Italy) and blot images were acquired by the chemiluminescence analyzer ChemiDoc™ Touch Imaging System (Bio-Rad Laboratories, Inc., Hercules, CA, USA). The optical density of target proteins was normalized to β-actin and expressed as mean ± SD.

### 4.4. RNA Isolation and Real-Time PCR

Total RNA was extracted from cells by using GenElute^™^ Mammalian Total RNA Miniprep Kit (Sigma-Aldrich Corporation, St. Louis, MI, USA) according to manufacturer’s instructions. cDNA targets were obtained from 2 µg of RNA by using SuperScript III Reverse Transcriptase (Thermo Fisher Scientific) and amplified with a 7300 Real-Time PCR System (Life Technologies, Milan, Italy) together with the reference cDNA of GAPDH by using probes marked with a 50-fluorescent dye (6-FAM) and a 30-quencher dye (NFQ). The detection of fluorescence emitted by samples required the use of ABI PRISM 7300-sequence detection system (Applied Biosystems, Waltham, MA, USA), and the quantitative analysis was performed comparing the threshold cycle (Ct) value of samples with that of the reference gene. The results were displayed in fold difference. The cDNA targets and primers are reported in Table 1. 

### 4.5. Immunofluorescence

A total of 1 × $10^{4}$ cells/well were plated in 24-multiwell plates containing sterile coverslips. Cells were fixed in 4% PFA for 15 min at room temperature and permeabilized with 0.1% Triton X-100 in PBS for 10 min at 37 °C. After blocking in goat serum 10% for 45 min at 37 °C (Sigma Aldrich, St. Louis, MO, USA), samples were incubated with anti-Tuj1 mouse monoclonal antibody (MoAb) (Santa Cruz Biotechnology, Inc., Milan, Italy), anti-NeuN mouse MoAb, anti-Neurofilament 68 mouse MoAb (Sigma Aldrich, St. Louis, MO, USA) and anti-GFAP mouse MoAb (Sigma Aldrich, St. Louis, MO, USA), at 1:100 dilution in goat serum 1.5% overnight at 4 °C. Specimens were washed and incubated with the secondary antibodies conjugated with the fluorescent probes FITC or TRITC (1:200) (Sigma Aldrich, St. Louis, MO, USA) in goat serum 1.5% for 1 h at 37 °C. To mark filamentous actin, glasses were incubated with the high-affinity filamentous actin (F-actin) probe conjugated to the green-fluorescent Alexa Fluor 488 dye, Alexa Fluor™ 488 Phalloidin 1x (Thermo Fisher Scientific Inc., Waltham, MA, USA), in a PBS solution supplemented with 1% goat serum 1.5% for 1 h at room temperature. Nuclei were stained with DAPI (Sigma Aldrich, St. Louis, MO, USA) 1 μg/mL for 30 min at 37 °C. Images were acquired with a ZEISS LSM 900 confocal microscope (ZEISS, Oberkochen, Germany). To minimize bleed-through, the excitation wavelength, the emission wavelength and power intensity were chosen in relation to each fluorophore.

For fluorescence intensity evaluation the fluorescence intensity was determined with the ImageJ software Fiji-2.14.0. The area of interest was selected to measure the relative fluorescence intensity. Data are expressed as percentage of respective controls.

To evaluate the population of NeuN^+^ cells to DAPI^+^, manual cell counting was performed by using the ImageJ multipoint tool Fiji-2.14.0. Only cells stained exclusively within nuclei for NeuN were counted. Data were expressed as percentage of relative controls.

### 4.6. In Vitro Tumorigenicity Assay

To assess the in vitro tumorigenicity, cell proliferation assay was performed [71]. DI-TNC1 cells were plated in triplicates at a density of 2 × $10^{5}$ and treated with lactoferrin and LPS as described above. As controls, DI-TNC1 and U-87MG cells were used and plated in triplicates at the same density. Cell count was performed on days 7, 9 and 12 post-treatment by using the Trypan Blue exclusion test. Non-viable cells were excluded from the cell count. Doubling time (Dt), expressed in hours, was calculated from the site www.doubling-time.com/compute.php (accessed on 10 October 2024) and specific growth rate (k) was determined from the following equation [72]:

(1) $$k=\frac{ln}{Dt}$$

where ln is the natural logarithm to base 2 and Dt is the doubling time.

### 4.7. Statistical Analysis

The statistical analysis was performed with RStudio Software R version 4.3.1 (R Foundation for Statistical Computing, Vienna, Austria). Data complying with ANOVA assumptions were analyzed with parametric analysis of variance followed by Fisher’s LSD post hoc test. In alternative, was performed Levene’s and Kruskal–Wallis tests followed by post hoc Tukey’s HSD test. We considered statistically different data with *p*-values < 0.05.

## Figures and Tables

**Table 1:** List of primers sequences.

cDNA Target	Sense Primer (5′–>3′)	Antisense Primer (3′–>5′)
TLR4	CGCTTTCACCTCTGCCTCACTACAG	ACACTACCACAATAACCTTCCGGCTC
GFAP	CACGAACGAGTCCCTAGAGC	GAAGAAAACCGCATCACCAT
IL-10	CTGTCATCGATTTCTCCCCTGT	CAGTAGATGCCGGGTGGTTC
SOX2	ACTAATCACAACAATCGCGGCGGC	GACGGGCGAAGTGCAATTGGA
NeuN	TTACGGAGCGGTCGTGTATC	GCAGGGTAAACCTTGGGACA
GAPDH	ACCACAGTCCCTGCCATCAG	TCCACCACCCTGTTGCTGTA

## Data Availability

Data are contained within the article.
